# Computational Opioid Prescribing: A Novel Application of Clinical Pharmacokinetics

**DOI:** 10.3109/15360288.2011.573527

**Published:** 2011-06-09

**Authors:** Oscar A Linares, Annemarie L Linares

**Keywords:** clinical pharmacokinetics, dose optimization, dose regimen design, dosing, opioids, pharmacokinetics

## Abstract

We implemented a pharmacokinetics-based mathematical modeling technique using algebra to assist pre-scribers with point-of-care opioid dosing. We call this technique computational opioid prescribing (COP). Because population pharmacokinetic parameter values are needed to estimate drug dosing regimen designs for individual patients using COP, and those values are not readily available to prescribers because they exist scattered in the vast pharmacology literature, we estimated the population pharmacokinetic parameter values for 12 commonly prescribed opioids from various sources using the bootstrap resampling technique. Our results show that opioid dosing regimen design, evaluation, and modification is feasible using COP. We conclude that COP is a new technique for the quantitative assessment of opioid dosing regimen design evaluation and adjustment, which may help prescribers to manage acute and chronic pain at the point-of-care. Potential benefits include opioid dose optimization and minimization of adverse opioid drug events, leading to potential improvement in patient treatment outcomes and safety.

## INTRODUCTION

The expected clinical outcome measure of interventional opioid pharmacotherapy for the treatment of acute and chronic pain is analgesia. However, studies indicate that many patients in pain are prescribed inadequate doses of opioid medications to relieve their pain (1–5). Multiple barriers to the adequate treatment of pain have been identified (6–9), but rational opioid dosing founded on pharmacokinetics-based opioid dosing regimens for individual patients has not been adequately addressed. To date, clinical pharmacokinetics-based opioid dosing regimen design and adjustment in acute and chronic pain management has been the subject of a limited number of publications (10, 11), although pharmacokinetics-based anesthetic opioid dosing is routine (12–14).

Opioid maximum concentrations (*C*_max_) and minimum concentrations (*C*_min_) may correspond to their minimum toxic concentrations (MTCs) and minimum effective concentrations (MECs). For example, the therapeutic range of morphine for analgesia is reported to be between 9.3 and 80 ng/mL, MEC range 9.3 to 23 ng/mL (15). This range implies that concentrations above 80 ng/mL are more likely to be associated with toxicity and concentrations below 9.3 ng/mL are more likely to produce little or no analgesic effect. Therefore, in the course of pain management with morphine, it is desirable that a dosage regimen for morphine produce plasma morphine concentrations within its therapeutic range. The goal of the design of an opioid dosing regimen is thus to achieve predicted plasma opioid concentrations within or at the boundaries of targeted *C*_max_ and *C*_min_ values or a desired target concentrations (*C*_target_).

This contribution introduces a novel application of clinical pharmacokinetics for opioid dosing regimen design and dose adjustment termed computational opioid prescribing (COP). A major aim of COP is the design of individualized opioid dosage regimens for patients in order to administer opioid doses based on their predicted plasma concentrations within preset *C*_max_ and *C*_min_ values or steady-state concentration (*C*_ss_) and *C*_target_ for multiple dosing regimens. Because pharmacokinetic parameter values for COP are not readily available, but rather exist scattered about in the vast pharmacology literature, we culled a large number of studies and applied the bootstrap resampling technique to compute opioid population pharmacokinetic parameter estimates for 12 opioids as reference for using COP.

## METHODS

### Opioid Population Pharmacokinetic Parameter Estimation

Population pharmacokinetic parameter values are often used to estimate drug dosing regimen designs for individual patients in whom patient-specific parameter values are not available (16, 17); however, opioid pharmacokinetic parameter values are scattered about in the vast pharmacology literature. Therefore, we culled a large number of studies using references (18–21), the reference lists therein, the reference lists of the references therein, and literature searches using PubMed.gov and Scholar.Google.com with search terms entered: opioids pharmacokinetics, opioids pharmacodynamics, and the latter terms, substituting the word opioids for each of the 12 individual opioids studied, so as to obtain pharmacokinetic parameter values for the following 12 opioids: (1) morphine, (2) tramadol, (3) codeine, (4) meperidine, (5), hydrocodone, (6) oxycodone immediate-release (IR), (7) oxycodone controlled-release (CR), (8) hydromorphone, (9) oxymorphone, (10) methadone, (11) fentanyl, and (12) buprenorphine. The inclusion criteria were human studies, articles published in the English language, any of the aforementioned 12 opioid drugs administered via intravenous, oral, or intramuscular routes, doses or range stating doses of the opiate(s) measured in plasma, opiate(s) concentration levels given in numerical or graphic form, and noncompartmental or compartmental pharmacokinetic parameters estimated. Necrokinetics data were exlcuded.

Since COP assumes a one-compartment model (see below), we pooled the reported values of the following four primary pharmacokinetic parameters of disposition for the one-compartment model: the half-life (*t*_1/2_), the first-order elimination rate constant (*k_e_*), the apparent volume of distribution (*V_d_*), and the systemic clearance rate (*C_l_*). These pharmacokinetic parameters are required to compute an appropriate opioid dose and dosing interval using the one-compartment model.

The large number of studies analyzed allowed a statistical approach in which the mean values from each study provided single data points (independent samples). When mean group values were not reported, the published experimental study data (22) was used to derive the primary pharmacokinetic parameter or parameters using modeling (23). All studies, despite their probable variable reliability, were accorded equal weight. Values for opioid bioavailability (*F*) were obtained from references (18–21), the references therein, or literature search. Values for the first-order absorption constant (*k_a_*) were obtained from references (18–21), the references therein, or literature search, but, when values were not available, they were computed using the equation:


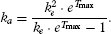
[1]

Opioid population pharmacokinetic parameter estimation using the bootstrap (see below) was not performed for *F* or *k_a_* values.

Opioid population pharmacokinetic parameter estimation was performed using the bootstrap resampling technique (24–27). The idea underpinning the bootstrap is to repeatedly sample with replacement (bootstrap samples) from the samples available, and use the bootstrap samples to compute the sampling distribution of the statistics of interest. Bootstrap samples look pretty much like drawing a new sample and provide good estimates of what would happen if we really were able to draw new fresh samples from the population of interest.

### Simplified Clinical Pharmacokinetic Equations for COP

A major aim of COP is to allow rapid, at the point-of-care, computations to be made of opioid clinical pharmacokinetic parameter values using pencil and paper or a calculator (28,29).

Equation 2 defines the target average steady-state plasma opioid concentration (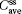) based on the minimum (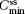) and the maximum (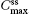) steady-state opioid therapeutic range concentrations:


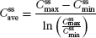
[2]

Equation 3 defines the opioid dosing rate (dose/τ), where τ represents the dosing interval necessary to achieve 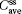. This calculation requires knowing the opioids *F* and *Cl*:


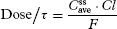
[3]

Equation 4 gives the rate of decline of plasma opioid concentration from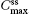 to 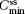:


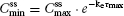
[4]

This rate is governed by the *t*_1/2_ or *k_e_* because *k_e_* = 0.693/*t*_1/2_ and vice-versa. Equation 4 can be solved for τ_max_, giving


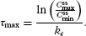
[5]

The opioid maintenance dose (*D_M_*) is given by


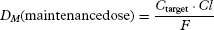
[6]

where *C*_target_ is the targeted plasma opioid concentration. In some cases, administration of a loading dose (*D_L_*) may be necessary:


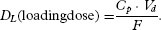
[7]

where *C_p_* represents the plasma opioid concentration. For multiple dosing, the accumulation ratio (AR), which represents the ratio of opioid in the body at steady-state relative to the amount of opioid in the body after a single dose, is given by


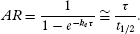
[8]

Hence, the maximum (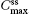) or peak plasma opioid concentration is given by


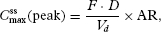
[9]

and the minimum (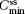) or trough plasma opioid concentration by


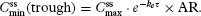
[10]

The average steady-state plasma opioid concentration (Equation 1) can also be obtained using


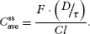
[11]

At any time (*T*), *C_p_* is given by


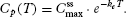
[12]

### Opioid Dosing Regimen Design Using COP

**FIGURE 1:**
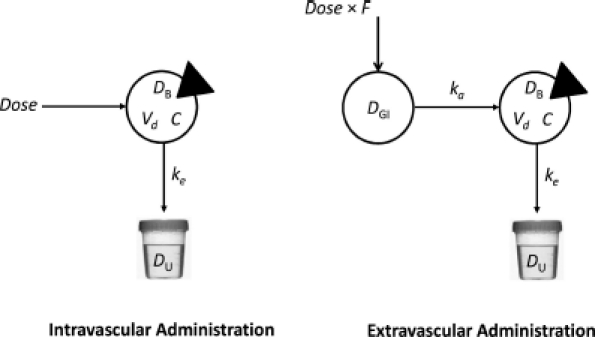
One-compartment models. The intravascular administration model (left panel) is characterized by a single sampled compartment into which a dose of drug is administered. The triangle cuts into the sampled compartment. In these models, *D_B_* represents the mass of drug in the body, *V_d_* is the apparent volume of distribution of drug, and *C* represents the drug concentration, equal to *D/V_d_*. *k_e_* represents the first-order elimination rate constant (h^−1^). Drug is eliminated in the urine unchanged and in metabolite form. In the extravascular administration model (right panel), a dose of drug is administered into a compartment from which the drug is transferred into the body via the gastrointestinal tract. *F* represents the drug's bioavailability. *k_a_* is the first-order absorption rate constant. The steady-state plasma concentration of opioid over time for oral dosing is independent of *k_a_*, so the path from *D_GI_* to *D_B_* equals *Dose* × *F*.

A major aim of COP is the design of an opioid dosage regimen that achieves predicted plasma opioid concentrations within a safe and effective range. COP assumes that opioid clinical pharmacokinetics can be reasonably approximated by a linear open one-compartment model with first-order absorption and first-order elimination (Figure (1)). This model has been shown to account for the pharmacokinetics of many important drugs (30), including the opioid analgesics (21). In certain circumstances, however, it may be associated with significant error in the calculation of a drug's absorption rate constant, *k_a_*, such as when *k_a_ = k_e_*.

The required data to design an opioid dosage regimen using COP is information about the pharmacokinetics of the opioid, the values reported in Table 1), and the opioid's therapeutic range (31). Pharmacokinetic parameter values calculated using COP can be expected to have an inter-individual variation of about 25%, which may be clinically acceptable.

**TABLE 1:** Opioid Population Pharmacokinetic Parameter Estimates Using the Bootstrap

Opioids (*N* = 1000)	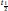	*k_e_* h^−1^	*V_d_* (L/kg)	*Cl* (L/h/kg)
Morphine	3.9 ± 1.7	0.318 ± 0.126	4.5 ± 1.4	1.43 ± 0.69
	(3.7, 4.0)	(0.305, 0.329)	(4.4, 4.6)	(1.35, 1.52)
Tramadol	5.5 ± 0.7	0.132 ± 0.017	2.8 ± 0.1	0.37 ± 0.06
	(5.4, 5.6)	(0.127, 0.136)	(2.7, 2.8)	(0.3598, 0.3763)
Codeine	2.6 ± 0.8	0.377 ± 0.116	3.0 ± 0.3	1.23 ± 0.45
	(2.5, 2.7)	(0.3653, 0.3880)	(2.9, 3.0)	(1.21, 1.26)
Meperidine	3.5 ± 0.9	0.242 ± 0.061	4.0 ± 0.1	0.98 ± 0.27
	(3.4, 3.6)	(0.234, 0.251)	(3.9, 4.0)	(0.97, 1.00)
Hydrocodone	6.1 ± 1.5	0.141 ± 0.036	4.0 ± 0.4	0.61 ± 0.20
	(6.0, 6.2)	(0.135, 0.148)	(3.9, 4.1)	(0.5938, 0.6230)
Oxycodone IR	4.5 ± 0.9	0.174 ± 0.034	2.8 ± 0.5	0.42 ± 0.003
	(4.4, 4.6)	(0.167, 0.180)	(2.7, 2.8)	(0.41, 0.42)
Oxycodone CR				
Phase I	33.5 ± 2.1 min	0.021 ± 0.001 min^−1^	2.8 ± 0.5	0.06 ± 0.01 L/min/kg
Phase II	(33.34, 33.66)	(0.0194, 0.0219)	(2.7, 2.8)	(0.055, 0.063)
	4.6 ± 0.6 h	0.160 ± 0.022 h^−1^	2.8 ± 0.6	0.58 ± 0.08 L/h/kg
	(4.5, 4.7)	(0.156, 0.165)	(2.7, 2.8)	(0.57, 0.59)
Hydromorphone	6.0 ± 1.7	0.154 ± 0.045	3.0 ± 0.6	0.54 ± 0.22
	(5.0, 6.1)	(0.147, 0.161)	(2.9, 3.1)	(0.52, 0.55)
Oxymorphone	8.0 ± 2.3	0.115 ± 0.033	3.0 ± 0.6	0.40 ± 0.17
	(7.8, 8.2)	(0.109, 0.122)	(2.9, 3.1)	(0.39, 0.42)
Methadone	35 ± 11.7	0.029 ± 0.009	5.5 ± 0.89	0.19 ± 0.08
	(34.6, 35.4)	(0.0258, 0.0323)	(5.4, 5.6)	(0.18, 0.20)
Fentanyl	7.5 ± 2.6	0.144 ± 0.049	5.5 ± 1.4	1.32 ± 0.73
	(7.3, 7.7)	(0.137, 0.152)	(5.4, 5.6)	(1.29, 1.35)
Buprenorphine	33.5 ± 8.7	0.026 ± 0.007	3.8 ± 1.5	0.13 ± 0.07
	(33.2, 33.8)	(0.023, 0.029)	(3.7, 4.0)	(0.12, 0.14)

*Values are bootstrap (49) mean ± SD; values in parentheses are bootstrap bias-corrected and -accelerated (50) 95% confidence intervals.

† All values computed from pooled literature values reported in the literature (see Methods) using the bootstrap (51) with *N* = 1000.

However, the following are assumptions and limitations of using the one-compartment model (32): (1) it is assumed that the pharmacokinetic parameters remain constant during the course of treatment; (2) changes in renal and/or hepatic function may pro-long the excretion of the fraction of opioid excreted unchanged in the urine, or metabolized by the liver; (3) congestive heart failure (CHF) and myocardial in farction (MI) may cause reduction in blood flow, resulting in reduced volume of distribution and clearance, thereby prolonging opioid elimination.

Qualifications for using the one-compartment model when a theoretically correct model is multi-compartmental are as follows (32,33): (1) the majority of the opioid distributes to rapidly perfused tissues and circulating fluids in the central compartment; and (2) although opioid distributes to tissues characterized by an open two-compartment model, since the equations are based on a one-compartment model, they can be applied to the open two-compartment model if instead of *k_e_*, the terminal rate constant, β, of the biexponential curve characterizing the open two-compartment model is used instead, and instead of the one-compartment model apparent volume of distribution, *V_d_*, the volume of the central compartment of the two-compartment model, *V_c_*, is used.

## RESULTS

Table 1) presents the population pharmacokinetic parameter estimates of the four primary pharmacokinetic parameters of disposition for the one-compartment model for the 12 opioids considered in this study. The fractional values for oral administration (unless otherwise indicated) of *F* for each of the opioids studied were as follows: *morphine* averaged 0.38 with a range of 0.15 to 0.64; *tramadol* was 0.73 males and 0.79 in females; *codeine* was 0.50; *meperidine* had a range of 0.40 to 0.60 with an average of 0.50; *hydrocodone* was 0.80; *oxycodone IR* had a range of 0.60 to 0.87 with an average of 0.74, which was similar for *oxycodone CR; hydromorphone* had a range of 0.52 to 0.58 with an average of 0.55; *oxymorphone* was 0.10; *methadone* was 0.80 with a range of 0.10 to 0.90; *fentanyl* was 0.33 for the oral preparation, 0.50 for the buccal, and 0.92 for the patch; and *buprenorphine* averaged 0.63 with a range of 0.50 to 0.75.

The *k_a_* values of each of the opioids studied were *morphine* 15.6 h^−1^; *tramadol* 2.6 h^−1^; *codeine* 15.6 h^−1^; *meperidine* 4.8 h^−1^; *hydrocodone* 2.8 h^−1^; *oxycodone IR* 3.5 h^−1^; *oxycodone CR* was 0.42 min^−1^ (first-phase release) and 3.2 h^−1^ (second-phase release); *hydromorphone* 3.1 h^−1^; *oxymorphone* 2.3 h^−1^; *methadone* 0.58 h^−1^; *fentanyl* 2.9 h^−1^; and *buprenorphine* 0.52 h^−1^.

In each of the following subsections, results using COP for opioid dosage regimen design and evaluation are demonstrated using morphine, because morphine is regarded as the benchmark of analgesics (34). The last case study, a forensic result, uses oxycodone IR. Morphine was used with the following pharmacokinetic characteristics (Table 1)): elimination half-life (*t*_1/2_) 3.9 hours; elimination rate constant (*k_e_*) 0.318 h^−1^; apparent volume of distribution (*V_d_*) 4.5 L/kg; clearance rate (*Cl*) 1.43 L/h/kg; *F* equal to 1.0 for intravenous dosing; *F* equal to 0.40 for oral dosing; and therapeutic range, 10 to 80 μg/L.

### Intravenous (IV) Bolus Dosing

Calculation of dose size and dosing interval is performed using the equations for maximum and minimum plasma drug levels at steady state, 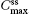 and 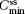, respectively. This method is called the limited fluctuation method or 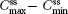 method (32). This approach is used when, within a dosing interval, the desired steady-state opioid plasma levels to achieve do not exceed *C*_max_ and do not undercut the desired *C*_min_.

First, we estimate a target average steady-state plasma morphine concentration (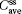) based on morphine's therapeutic window. Equation 1 defines 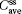 based on the minimum (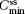) and maximum (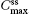) steady-state morphine therapeutic window concentrations equal to 10 and 80 μL, respectively:


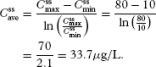
[13]

Note that 33.7 μg/L is slightly different than the algebraic average of 35 μg/L because we have assumed that morphine pharmacokinetics can be described by a one-compartment model. Therefore, the plasma concentration of morphine undergoes first-order elimination corresponding to exponential decline instead of simple linear disposition (35).

Next, we estimate the dosing rate (dose/τ), where r represents the dosing interval necessary to achieve 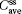. This calculation requires knowing *F* (equal to 1 for IV dosing) and *Cl* of morphine (Table 1)):


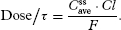
[14]

In the United States, where the average male weighs 86.6 kg (190.9 lbs) and the average female weighs 74.4 kg (164.0 lbs) (36), the estimates of morphine population *Cl* are 123.8 L/h for males and 106.4 L/h for females. For IV administration, morphine's *F* is equal to 1. Hence, for the average male,


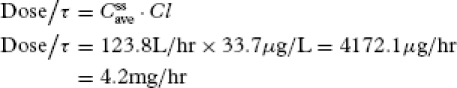
[15]

and for the average female,


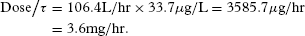
[16]

Now, we estimate the maximum dosing interval τ_max_. The rate of decline of the plasma morphine concentration from 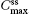 to 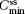 is governed by morphine's *t*_1/2_ or *k_e_*. So, we can estimate how long it would take for the plasma morphine concentration to decline from a maximum to a minimum level. Setting morphine's therapeutic window boundaries (10 to 80 μg/L) as the limits of 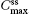 and 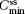, the time it takes for the plasma morphine concentration to fall from 80 to 10 μg/L. can be estimated as follows:


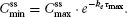
[17]

This equation can be solved forτ_max_, giving:


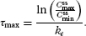
[18]

Substituting for morphine's *k_e_* in Equation 18 gives,


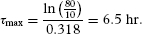
[19]

The τ_max_ of 6.5 h means that the longest dosing interval that may be selected for the patient's dosing is 6.5 hours. After that time, morphine levels will fall below 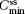. Also, because morphine administration every 6.5 hours is not practical, choose a τ from one of the following practical dosing intervals: 2, 4, 6, 8, 12, or 24 hours. For convenience, one might be tempted to choose a longer τ, e.g., 8 hours, however, a practical τ cannot be greater than τ_max_ if the desired outcome is to maintain plasma morphine concentrations between 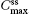
and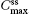. In this case, a τ of 6 hours is a good first choice.

Knowing the dosing interval and the dose rate (Dose/τ), the maintenance dose (*D_M_*) for the average male is given by:


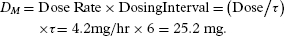
[20]

A similar computation may be made to determine *D_M_* for the average female. If the dose is not practical or the available strengths of the drug do not allow administration of the calculated dose, the dose may be rounded to the nearest practical number. For instance, the above morphine dose may be rounded to 25 mg.

### Case Study 1

The patient is a 55-year-old Caucasian man newly diagnosed with cancer of the prostate and metastases to the spine with normal cardiac, hepatic, and renal function. He is experiencing 10/10 back pain. The nurse on duty caring for the patient informs the doctor that the patient has not received morphine in the past, so he is opioid naive. The nurse is not comfortable administering intravenous morphine 25 mg every 6 hours to the patient. The doctor searches the literature and finds evidence that in cancer patients, morphine levels at or above 20 μg/L are considered to be analgesic in most patients (37). This value is about 60% of the plasma morphine concentration calculated using COP (Equation 13). Using COP, the doctor simulates a morphine dosage regimen equal to 12 mg every 6 hours to assess the effect it would have on morphine pharmacokinetics in this patient.

The doctor reestimates 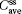 to predict the new plasma morphine concentration using the practical τ of 6 hours and the intravenous morphine dose of 12 mg.


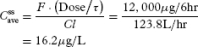
[21]

The simulation predicts that cutting the morphine dose to 12 mg would not achieve the evidence-based steady-state analgesic plasma morphine concentration of 20 μg/L. or more. The doctor performs another simulation using an intravenous morphine dose of 15 mg every 6 hours.


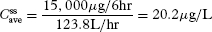
[22]

The selected dose now predicts a plasma morphine concentration within the evidence-based analgesic value. To estimate the *C*_max_ and *C*_min_ values with the reduced dose regimen, first, the *C*_max_ and *C*_min_ after the first dose (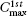
and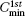, respectively) are estimated. Then, using the accumulation ratio (AR), the 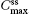 and 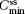 values are computed as follows:


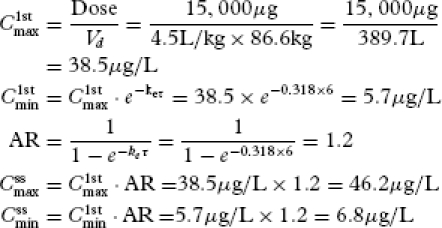
[23]

AR represents the ratio of opioid in the body at steady-state relative to the amount of opioid in the body after a single dose (35). Thus, a dosage regimen of 15 mg intravenous morphine every 6 hours would be expected to result in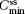, 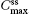, and 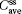
values of 6.8, 46.2, and 20.2 μg/L, respectively. These results suggest that, due to fluctuations in plasma morphine concentrations, the patient's pain may be undertreated towards the latter third of the dosing interval. COP indicates that special clinical attention to the patient's symptoms should be made at around that time.

In some cases, administration of a loading dose (*D_L_*) or starting dose (*D_S_*) may be necessary, particularly if the half-life of the opioid is long and/or rapid achievement of therapeutic opioid concentrations is important, e.g., in acute pain. In these cases, *D_L_* may be calculated using the *t*_1/2_ by the following method:


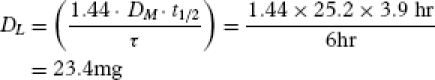
[24]

The calculated dose should be adjusted for administration based on available morphine strengths.

### Extravascular (Oral) Dosing

The computation of dose and dosing interval after extravascular dosing (e.g., oral administration) is slightly more complicated than after intravenous (IV) bolus dosing because the absorption rate (*k_a_*) and *F* are important factors, in addition to the other four basic clinical pharmacokinetic parameters. Nevertheless, simplified clinical pharmacokinetic-based equations for COP allow easy calculation of oral dosage regimen designs (see above) that do not require *k_a_*, because it can be shown that the terminal equilibrium level under constant infusion equals the average blood level over time for the terminal steady-state under multiple IV or oral dosing (38). Also, a simplification which applies to opioids, is when the *k_a_* is much faster than the *k_e_*. Under this condition, *k_a_* may be assumed to be instantaneous for practical purposes. In general, if the *k_a_* is 5 to 7 times greater than the *k_e_*, the absorption rate may be assumed to be instantaneous. This circumstance is similar to the IV bolus administration of opioid (39), but with *F* less than 1 so that the equations for *IV* bolus dosing apply. Therefore, the equations used for IV bolus dosing above can also be used to design extravascular (oral) dosage regimens with reasonable accuracy.

### Case Study 2

The patient is a 52-year-old African American woman weighing 302 lbs (137 kg), and is 2 days postoperative from right knee arthroplasty. She complains of 8/10 pain. She requests oral pain medication. Using the opioid plasma target concentration method, the doctor selects a target morphine plasma concentration equal to half of its MTC, i.e., 40 μg/L. *Fis* set equal to its algebraic average, which is calculated as (0.15+ 0.64)/2 = 0.40. Then, the morphine dose for starting treatment (*D_S_* = *D_L_*) is calculated using the following equation:


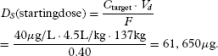
[25]

Multiplying by 0.001 converts μg to mg, so the starting dose equals 62 mg, which can be rounded to 60 mg for administration.

The time to reach steady state for morphine is *T*_ss_ = 5 × *t*_1/2_ = 20 hours. By this time, it can be assumed that morphine's *rate in* = *rate out*. Thus, the morphine maintenance dose to achieve a steady state in *T*_ss_ is given by


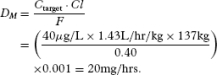
[26]

Now, selecting a dosing interval where τ = 6 hours gives *D_M_* = 120 mg every 6 hours. This is the dose required to maintain a steady-state plasma concentration of morphine of 40 μg/L. We confirm the dose as follows.

Compute the accumulation ratio using AR = τ / *t*_1/2_ = 6/4 = 1.5, then


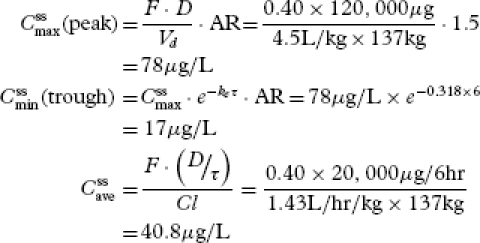
[27]

Thus, COP demonstrates that our oral morphine dosage regimen design achieved the therapeutic goal of maintaining a steady-state plasma morphine concentration of 40 μg/L. The calculated value is slightly higher due to computer round off error. Moreover, COP shows that plasma morphine fluctuations about 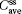 remain close to the bounds of morphine's therapeutic window for analgesia. However, towards the end of the dosing interval, the concentration falls slightly below the evidence-based plasma morphine concentration for analgesia of 20 μg/L. COP indicates that an adjustment in the dosing interval can be made if the patient complains of pain towards the end of the dosing interval. For example, using Equations 12 or 17, at the 5th-hour into the 6th-hour dosing interval, plasma morphine concentration is 24 μg/L, indicating that the patient needs to be followed closely for symptoms of pain during the last hour of the dosing interval when plasma morphine concentrations fall below the evidence-based analgesic concentration.

### Constant Intravenous Infusion

To determine intravenous infusion rate we need only determine the infusion rate constant (*k*_0_) and do not need to compute τ. To determine a constant rate intravenous infusion of morphine, we estimate *k*_0_ based on a desired morphine *C*_target_ to achieve, and its *Cl*, so that *k*_0_ = *C*_target_ · *Cl*. We chose a value for *C*_target_ within morphine's therapeutic window. If rapid achievement of steady-state morphine concentration is desired, an intravenous loading dose may be calculated using Equation 7 or simply *D_L_* = *C*_target_ · *V_d_*. For example, an intravenous *D_L_* of morphine based on *C*_target_ = 25 μg/L in an average-weight woman in the United State equals 25 μg/L × 4.5 L/kg × 74.4 kg × 0.001 = 8 mg. Now, *k*_0_ = 25 μg/L × 1.43 L/h/kg × 74.4 kg × 0.001 = 3 mg/h. Thus, administration of *D_L_* produces a plasma morphine concentration of about 24 μg/L (not exact due to computer round-off error), which is maintained by simultaneously starting a morphine infusion at a rate of 3 mg/h.

### Opioid Dosing Regimen Evaluation

Dosing regimen evaluation using COP applies to patients who present on a dosage regimen prescribed by another prescriber or an emergency department (ED) physician. The goal is to determine the adequacy of the regimen to achieve evidence-based analgesic plasma concentrations of opioid.

### Case Study 3

A 38-year-old average weight man was seen in the ED 24 hours ago and placed on an oral regimen of 10 mg morphine sulfate every 8 h for 7/10 back pain secondary to a fall from a 6-foot ladder. He was instructed to follow-up with pain management on the next day. He presented 24 hours later. On evaluation, he reported taking medications as prescribed and a moderate increase in pain which he rated 8/10. Lumbosacral, spine, and hip films in the ER were normal. At the discharge dose and dosing interval, it would take 20 hours to reach a steady-state plasma morphine concentration of


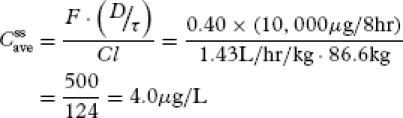
[28]

Clearly, the patients dosage regimen is inadequate to meet his analgesic needs, since only morphine levels at or above 20 μg/L are considered to be analgesic (15,37). Thus, a dosage adjustment, rather than an extensive work-up, was indicated to manage this patients increasing pain.

### Case Study 4

A petite 29-year-old woman weighing 110 lbs (50 kg) fell from a chair and fractured her coccyx. She was seen in the ED and started on oral morphine sulfate 20 mg every 4 hours. She presented for pain management within 24 hours. She reported increasing pain. On direct questioning, she reported that when she took the first dose of morphine, she felt relief for about 15 minutes. The doctor performed a clinical pharmacokinetics assessment to determine 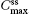, 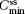, and 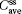 as follows:


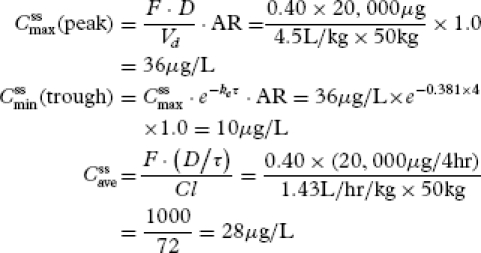
[29]

The patient's clinical pharmacokinetics profile looks good, there is no evidence that she is receiving too much morphine. Her results show that predicted trough levels are at the lower end of the therapeutic window.

To design an individualized dosage regimen based on the patients response to the morphine, the doctor predicts what her plasma concentration of morphine would be 15 minutes after she takes a dose:


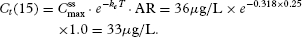
[30]

Based on this computation, the doctor decides to set *C*_target_ = 35 μg/L, which is the average value between 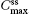
and *C_t_*(30), to calculate a maintenance dose:


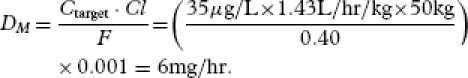
[31]

Since 24 mg every 6 hours is not practical, the doctor selects a dose of 25 mg every 4 hours for the patient. This dose will be associated with a 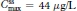 and 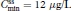. One hour after a dose, the plasma morphine concentration will be 32 μg/L. By 1 hour and 15 minutes after a dose, plasma morphine concentration will be 30 μg/L, but by 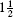 hours it will have fallen to 28 μg/L.

The patient is seen for follow-up within 3 days. She reports being pain free up for about 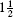 to 2 hours after taking a scheduled morphine dose. Thus, COP predicts she requires a plasma morphine concentration ≥30 μg/L for pain relief. In addition, COP indicates the patient will need a long-acting morphine combined with rescue doses of short-acting morphine for adequate pain control.

### Case Study 5

In the course of chronic pain management, if a patient with chronic pain dies and becomes a medical examiners case, opioid levels obtained at autopsy can be misinterpreted in determining cause of death (40). In February 1999, a physician was charged with three counts of murder alleged to result from lethal oxycodone doses (41). In one case, the victim died in a motor vehicle accident, but at autopsy was found to have an oxycodone blood level of 21,900 μg/L. Assuming no significant postmortem redistribution of oxycodone, using the oxycodone IR values in Table 1) with *F* = 0.74,


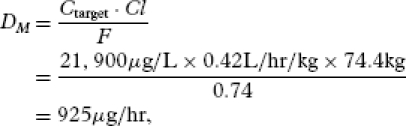
[32]

which for dosing intervals equal to every 4 and 6 hours, the corresponding administered doses of oxycodone IR would equal 3700 μg (4 mg) and 5500 μg (6 mg) every 4 or 6 hours, respectively. COP indicates that an oxycodone blood level of 21,900 μg/L can be associated with low-dose oxycodone IR administration, not lethal doses, as implied by the County Medical Examiner in this case.

In this case, the patient's boyfriend was driving the car. The patient died in the motor vehicle accident of a hangman's fracture of the cervical spine, open skull fracture, and severe internal injuries. Because she had opioid drugs in her system, the County Medical Examiner determined that she died of an opioid overdose instead of the obvious lethal injuries sustained in the motor vehicle accident (42).

## DISCUSSION

Prescribers, in general, may be concerned that the mathematics of pharmacokinetics precludes practical application at the point-of-care. This opinion may be rooted in an assumption that if a drug is not working, more should be given or, conversely, if it is producing toxicity, less should be given. This empirical approach, although not without merit, means that learning how to use any opioid safely will entail a long an inefficient period of trial and error. Indeed, we are presently witnessing a global epidemic of opioid-related deaths due to overdose (43,44). These opioid-related increases in deaths may be due, in part, to an empirical approach to opioid dosing regimen design and modification. COP may therefore constitute a potentially important tool for opioid dosing regimen design and adjustment, which may lead to the prevention of adverse opioid drug effects, including death from iatrogenic overdose.

For example, in *U.S. v. Hurwitz* (45), an expert witness for the government testified that high-dose opioid therapy typically involved doses of the equivalent of approximately 195 mg of morphine a day. Our results indicate that the average man would receive about 100 mg of morphine per day. The doses prescribed by Hurwitz, however, vastly exceeded those quantities. Hurwitz often wrote prescriptions calling for a patient to take 30 80-mg OxyContin tablets per day, which is the equivalent of a total daily dose of 2,400,000 μg. Using COP, such a dose would be associated with a plasma oxycodone concentration of about 9898 μg/L. Plasma oxycodone concentration range from 600 to 5000 μg/L is associated with induction of coma and death (46).

A potential limitation of the current implementation of COP is that it is not integrated into an opioid therapeutic drug monitoring (TDM) pharmacovigilance program, TDM is not routinely used in everyday clinical practice when prescribing opi-oids, as its role in opioid management is not yet well defined. Nevertheless, indications for TDM are relevant for all drugs, with or without validated therapeutic windows (47). When literature references on therapeutic ranges are not available, targeted concentration ranges should be plasma concentrations that have been observed at therapeutic doses of the drug.

We cannot expect that measured plasma opioid drug concentrations will be identical to opioid drug concentration predicted using COP, because there is always inter- and intraindividual variation, measurement error, and model misspecification error. Mis-specification error is due to representing the body as a single compartment. Thus, the difference between the patient and the model (interindividual variability), and the difference between the patient's “true” value, if it could be known, and the measured concentration (residual intraindividual variability) will account for the differences in the COP predictions relative to the measured drug concentration.

The authors of this contribution have made every effort to ensure the accuracy of the information provided at the time of its composition. Nevertheless, it remains the responsibility of every prescriber to evaluate the appropriateness of a particular result in the context of the actual clinical situation and to consider any new developments in the field. Although the authors have been careful to make COP adhere to current standards and responsible literature, the authors recommend prescribers consult appropriate informational sources when prescribing new or unfamiliar opioid drugs.

COP was designed as a clinical-decision support tool for prescribers at the point-of-care to provide guidance for individualizing opioid drug therapy. Although COP may be used by prescribers for dosage regimen analysis, design, and modification, *caution* should be exercised when applying COP results. The large between–individual patient variability in responses to opioid drug administration suggests that COP will not work accurately in all cases. Uncertainty in patient opioid dosage histories and compliance with opioid dosage regimens makes all efforts at opioid dosage regimen analysis tentative at best. Moreover, the expected variability and occasional errors in the laboratory may confound proper interpretation. These sources of variability underscore the importance of using COP in conjunction with a comprehensive patient evaluation and management approach. This includes careful attention to correct diagnosis; identification of therapeutic goals and objective therapeutic end points; correct choice of pain relievers; constant assessment and reassessment of therapeutic outcomes; and, when appropriate, measurement of urine drug levels for therapeutic drug monitoring. Proper interpretation of analytical data requires not only an understanding of the circumstances surrounding a case but also an appreciation of the circumstances under which any data cited to aid interpretation were produced (48).

## CONCLUSION

COP is a novel application of clinical pharmacokinet-ics to opioid dosing regimen design, evaluation, and modification. COP is a quantitative clinical-decision support tool providing guidance for individualizing opioid drug therapy at the point-of-care. Application of COP may prevent iatrogenic overdoses of opioids and it may reduce prescription costs by allowing patient dosing regimens to be individualized. Finally, COP may be useful in drug abuse prevention and detection (DAPD), when it is used in conjunction with a program of quantitative urine drug monitoring, as it can allow quantification of expected urine excretion of administered opioids.

## FUTURE DIRECTIONS

We are currently developing software for Apple's iOS platform for point-of-care COP. We are also working on pharmacokinetic phenotyping of individual-based opioid pharmacokinetics and incorporating pharmacogenomics information into *k_e_*—pharmacokinetics and pharmacogenomics modeling.

## References

[1] Bonham V (2001). Race, ethnicity, and pain treatment: striving to understand the causes and solutions to the disparities in pain treatment. J Law Med Ethics..

[2] Furrow B (2001). Pain management and provider liability: no more excuses. J Law Med Ethics..

[3] Hoffman D, Tarzian A (2001). The girl who cried paa bias against women in the treatment of pain. J Law Med Ethics..

[4] Johnson S (2001). Relieving unnessary, treatable pain for the sake of human diginity. J Law Med Ethics..

[5] Deandrea S, Montanari M, Moja L, Apolone G (2008). Prevalence of undertreatment in cancer pain. A review of published literature. Ann Oncol..

[6] Drayer R, Henderson J, Reidenberg M (1999). Barriers to better pain control in hospitalized patients. J Pain Symptom Manag.

[7] Glajcben M (2001). Chronic patreatment barriers and strategies for clinical practice. JABFP.

[8] Apfelbaum J, Chen C, Mehta S, Gan T (2003). Postoperative pain experience: results from a National survey suggest postoperative pain continues to be undermanages. Anesth Analg..

[9] Richard J, Reidenberg M (2005). The risk of disciplinary action by State Medical Boards against physicians prescribing opioids. J Pain Symptom Manag..

[10] Upton R, Semple T, Mcintyre P (1997). Pharmacokinetic optimization of opioid treatment in acute pain therapy. Clin Pharmacokinet..

[11] Linares O (2010). Pharmacokinetics-based opioid prescribing: case report. Pain Pract..

[12] Mather L (1987). Opioid pharmacokinetics in relation to their effects. Anaesth Intensive Care..

[13] Shafer S, Varvel J (1991). Pharmacokinetics, pharmacodynamics, and rational opioid selection. Anesthesiology..

[14] Maitre P (1992). Opioid pharmacokinetics: relationship to response. Curr Opin Anaesthesiol..

[15] Gourlay G, Willis R, Lamberty J (1986). A double-blind comparison of the efficacy of methadone and morphine in postoperative pain control. Anesthesiology..

[16] Ette E, Williams P, Lane J (2004). Population pharmacokinetics III: design, analysis, and application of population pharmacokinetics. Ann Pharmacother..

[17] Nehvar R (2006). Estimation of pharmacokinetic parameters based on the patient-adjusted population data. Am J Pharmaceut Educ..

[18] Karch S (2007). Pharmacokinetics and Pharmacodynamics of Abused Drugs.

[19] Karch S (2007). Drug Abuse Handbook.

[20] Karch S (2008). Karch's Pathology of Drug Abuse.

[21] Baselt R (2009). Disposition of Toxic Drugs and Chemicals in Man.

[22] Jacob H (1984). Using Published Data: Errors and Remedies.

[23] Wastney M, Patterson B, Linares O, Greif P, Boston R (1999). Investigating Biological Systems Using Modeling: Strategies and Software.

[24] Efron B (1979). Bootstrap methods: Another look at the jacknife. Ann Stat..

[25] Efron B (2003). Second thoughts on the bootstrap. Stat Sci..

[26] Yafune A, Ishiguro M (1999). Bootstrap approach for constructing confidence intervals for population pharmacokinetic parameters. I: a use of bootstrap standard error. Stat Med..

[27] Yafune A, Ishiguro M (1999). Bootstrap approach for constructing confidence intervals for population pharmacokinetic parameters. II: a bootstrap modification of standard two-stage (STS) method for phase I trial. Stat Med..

[28] Perlin E, Taylor R, Peck C (1985). Clinical pharmacokinetics: a simplified approach, part 1. J Natl Med Assoc..

[29] Perlin E, Varvel J, Peck C (1986). Clinical pharmacokinetics: a simplified approach, part 2. J Natl Med Assoc..

[30] Dvorchik B, Vesell E (1978). Significance of error associated with use of the one-compartment formula to calculate clearance of thirty-eight drugs. Clin Pharmacol Ther..

[31] Mehvar R (2006). Estimation of pharmacokinetic parameters based on patient-adjusted population data. Am J Pharmaceut Educ..

[32] Ritschel W, Kearns G (2009). Handbook of Basic Pharmacokinetics.

[33] Schoenwald R (2001). Pharmacokinetic Principles of Dosing Adjustment.

[34] Armstrong S, Cozza K (2003). Pharmacokinetic drug interactions of morphine, codeine, and their derivatives: theory and clinical reality. Psychosomatics..

[35] Mehvar R (1998). Pharmacokinetic-based design and modification of dosage regimens. Am J Pharmaceut Educ..

[36] Ogden C, Fryar C, Carroll M, Flegal K (2004). Mean Body Weight, Height, and Body Mass Index in the United States 1960–2002. Advanced Data From Vital and Health Statistics; No. 347. Hyattsville. MD: National Center for Health Statistics.

[37] Sawe J (1981). Morphine kinetics in cancer patients. Clin Pharm Ther..

[38] Arnold D, Grics H, Krewski D (1990). Handbook of in Vivo Toxicity Testing.

[39] Hacker M, Bachmann K, Meser W (2009). Pharmacology: Principles and Practice.

[40] Jung B, Reidenberg M (2005). Interpretation of opioid levels: comparison of levels during chronic pain therapy to levels from forensic autopsies. Clin Pharmacol Therapeut..

[41] Meier B (2002). OxyContin prescribers face charges in fatal overdoses. The New York Times. January 19, 2002.

[42] Flannery J (2006). Pain in America—And How Our Government Makes it Worse.

[43] Spiehler V (1989). Computer-assisted interpretation in forensic toxicology: morphine-involved deaths. J Forensic Sci..

[44] Paulozzi L, Ryan G (2006). Opioid analgesics and rates of fatal drug poisonong in the United States. Am J Prev Med..

[45] Unites States v. Hurwitz, 459 F.3d 463 (4th Cir. 2006) (2006).

[46] Schulz M, Schimoldt A (2003). Therapeutic and toxic blood concentration of more than 800 drugs and xenobiotics. Pharmazie..

[47] Baumann P, Hiemke C, Ulrich S, Eckerman G, Gaertner I, Gerlach M (2004). The AGNP-TDM Expert Group Consensus Guidelines: therapeutic drug monitoring in psychiatry. Pharmacopsychiatry..

[48] Peck C, Conner D, Murphy M (1991). Bedside Clinical Pharmacokinetics: Simple Techniques for Individualizing Drug Therapy.

[49] Efron B, Tibshirani R (1986). Bootstrap measures for standard errors, confidence intervals, and other measures of statistical accuracy. Stat Sci..

[50] Efron B (1987). Better bootstrap confidence intervals (with discussion). J Am Stat Assoc..

[51] Efron B, Tibshirani R (1986). An Introduction to the Bootstrap.

